# Deuterium brain imaging at 7T during D_2_O dosing

**DOI:** 10.1002/mrm.29539

**Published:** 2022-11-25

**Authors:** Daniel Cocking, Robin A. Damion, Hester Franks, Matthew Jaconelli, Daniel Wilkinson, Matthew Brook, Dorothee P. Auer, Richard Bowtell

**Affiliations:** ^1^ School of Physics and Astronomy University of Nottingham Nottingham UK; ^2^ Sir Peter Mansfield Imaging Centre University of Nottingham Nottingham UK; ^3^ Mental Health and Clinical Neuroscience, School of Medicine University of Nottingham Nottingham UK; ^4^ NIHR Nottingham Biomedical Research Centre/Nottingham Clinical Research Facilities Queen's Medical Centre Nottingham UK; ^5^ Centre for Cancer Sciences Biodiscovery Institute, School of Medicine University of Nottingham Nottingham UK; ^6^ Department of Oncology Nottingham University Hospitals NHS Trust Nottingham UK; ^7^ MRC‐Versus Arthritis Centre for Musculoskeletal Ageing Research University of Nottingham Nottingham UK; ^8^ School of Life Sciences University of Nottingham Nottingham UK; ^9^ Division of Medical Sciences and Graduate Entry Medicine School of Medicine, University of Nottingham Nottingham UK

**Keywords:** D_2_O loading, deuterium (^2^H), heavy water, human brain, MEGE, relaxation times

## Abstract

**Purpose:**

To characterize the (^2^H) deuterium MR signal measured from human brain at 7T in participants loading with D_2_O to ˜1.5% enrichment over a six‐week period.

**Methods:**

^2^H spectroscopy and imaging measurements were used to track the time‐course of ^2^H enrichment within the brain during the initial eight‐hour loading period in two participants. Multi‐echo gradient echo (MEGE) images were acquired at a range of TR values from four participants during the steady‐state loading period and used for mapping ^2^H T_1_ and T_2_^*^ relaxation times. Co‐registration to higher resolution ^1^H images allowed T_1_ and T_2_^*^ relaxation times of deuterium in HDO in cerebrospinal fluid (CSF), gray matter (GM), and white matter (WM) to be estimated.

**Results:**

^2^H concentrations measured during the eight‐hour loading were consistent with values estimated from cumulative D_2_O dose and body mass. Signal changes measured from three different regions of the brain during loading showed similar time‐courses. After summing over echoes, gradient echo brain images acquired in 7.5 minutes with a voxel volume of 0.36 ml showed an SNR of ˜16 in subjects loaded to 1.5%. T_1_‐values for deuterium in HDO were significantly shorter than corresponding values for ^1^H in H_2_O, while T_2_^*^ values were similar. ^2^H relaxation times in CSF were significantly longer than in GM or WM.

**Conclusion:**

Deuterium MR Measurements at 7T were used to track the increase in concentration of ^2^H in brain during heavy water loading. ^2^H T_1_ and T_2_^*^ relaxation times from water in GM, WM, and CSF are reported.

## 1 INTRODUCTION

The low natural abundance (˜0.015%) and gyromagnetic ratio (6.54 MHz/T) of deuterium (^2^H) reduce the available NMR signal compared to ^1^H. However, the shorter longitudinal relaxation times of ^2^H allow faster signal averaging, partially compensating for the reduction in SNR associated with the reduced signal strength. The minimal equipment modifications required for implementing ^2^H imaging and the simplicity of the pulse sequences that can be used, mean that ^2^H imaging has significant potential for use in clinical applications. This has led to an increasing interest in the use of deuterium magnetic resonance in conjunction with injection or ingestion of ^2^H‐labeled compounds, as a means of monitoring cellular metabolism.^1^, ^2^, ^3^

Deuterium metabolic imaging (DMI) involves using ^2^H chemical shift imaging to map the distribution of the metabolic products of administered ^2^H‐labeled compounds. The majority of experiments in humans and animals have used [6,6′‐D_2_] glucose.^2^, ^3^, ^4^, ^5^, ^6^, ^7^, ^8^, ^9^, ^10^, ^11^, ^12^, ^13^, ^14^, ^15^, ^16^ In this case, lactate and glutamine/glutamate (Glx) are produced alongside deuterated water (HDO), and their relative concentrations reflect the cells' preference for glucose metabolism, i.e., aerobic, anaerobic glycolysis or oxidative phosphorylation. Spatially localized, elevated, lactate production has been observed using DMI in a patient with a glioblastoma,^2^ in keeping with an increased glycolysis in neoplastic cells, known as the Warburg effect.^17^

DMI can provide spatially resolved measurements of metabolite concentrations and pathway fluxes,^1^, ^4^ but this often requires knowledge of the local relaxation times of the ^2^H signals from metabolites. The signal from naturally abundant HDO can be used in calculating absolute concentrations of other metabolites. Therefore, knowledge of the relaxation times of HDO in different tissues is important for quantitative measurements. Previous measurements of HDO relaxation times in human participants have used non‐localized signals^2^, ^3^, ^11^ which do not allow the variation of relaxation times across regions and compartments to be evaluated.

Oral intake of heavy water is commonly used for assessment of body composition^18^ and is increasingly being applied in studies of triglyceride synthesis^19^ and protein turnover.^20^, ^21^, ^22^ These approaches generally involve analysis of body fluid samples or tissue biopsies. However, ^2^H magnetic resonance allows direct, non‐invasive, measurement of the concentration of HDO, deuterated lipids, and other metabolic products of ^2^H labeled water and so could complement invasive measurements. The feasibility of imaging the distribution of HDO in the body following oral ingestion of D_2_O was demonstrated in animal experiments carried out in the 1980 s,^23^, ^24^, ^25^, ^26^, ^27^ but has not yet been performed in humans.

Here, we implemented deuterium MRI at 7T and used it to characterize HDO signals from the human brain in four healthy participants who increased their deuterated water content to ˜1.5% for a six‐week period by drinking D_2_O. The heavy water loading was carried out as part of a parallel study.

## 2 METHODS

Six healthy participants took part in this ^2^H^‐^imaging sub‐study which was approved by the local institutional ethics committee with the volunteers giving informed consent. Two participants were scanned during a set‐up phase in which we established the feasibility of ^2^H imaging and identified favorable imaging parameters. Here, we report data from four participants (A‐D) who were subsequently scanned using the optimized imaging protocols on a 7T Achieva scanner (Philips Healthcare), operating at 45.8 MHz for ^2^H. A 26.4‐cm‐inner‐diameter, dual‐tuned ^1^H/^2^H birdcage coil (Rapid Biomedical) was used for deuterium measurements, while the standard, 32‐channel Rx/2‐channel Tx head coil (Nova Medical) was used for acquiring anatomical ^1^H images.

The parallel study required an initial loading regime in which the targeted enrichment was built up in around eight hours. This involved the participants drinking between 12 and 16, ˜50 ml doses of 70% D_2_O/30% H_2_O (one dose every ˜30 minutes), with the total amount of D_2_O consumed adjusted according to the participant's body weight to produce 1.5% enrichment. Participants subsequently drank ˜50 ml of D_2_O each morning over the six‐week study period to maintain 1.5% enrichment. Similar enrichment levels and durations have been used in recent studies^28^, ^29^, ^30^ with no adverse events reported, however some participants experienced a brief period of dizziness during the initial loading phase due to the rapid rise in body water enrichment.^28^ Saliva samples were collected from participants at regular intervals during the study and analyzed using gas chromatography–mass spectrometry (GCMS).^31^

Two participants (A and B) were scanned during the initial eight‐hour loading period to monitor the time‐course of deuterated water concentration changes in the brain. A scanning protocol of ˜15 minutes duration was performed before dosing and after 30, 90, 150, 210, 270, 360, 420, and 540 minutes. The protocol comprised a ^1^H scout scan for planning, followed by acquisition of ^2^H pulse‐acquire spectra from the whole head and from a 2‐cm‐thick axial slice positioned over the lateral ventricles. Both used the following scan parameters: flip angle α = 90°, 2048 samples, bandwidth (BW) = 3000 Hz, repetition time TR = 1 s an 64 averages (scan time, T_scan_ = 64 s). We then acquired axial, 3D MEGE ^2^H images (20 averages, T_scan_ = 453 s, FOV = 288 × 288× 80 mm^3^, 6 × 6 × 10 mm^3^ voxels, α = 33°, TR = 62 ms, five echoes, TE_1_ = 8.9 ms and ΔTE = 8.4 ms). Axial ^1^H 3D GE images (T_scan_ = 232 s, 32 slices, FOV = 288 × 288 × 80 mm^3^, 3 × 3 × 2.5 mm^3^ voxels, TE = 5.9 ms, TR = 39 ms) were also acquired. This scanning protocol was repeated 17 days after the initial loading to provide comparative data at steady‐state enrichment. The spectroscopy measurements made before loading provided an estimate of the signal from naturally abundant deuterium in water: scaling subsequent measurements then allowed the absolute HDO concentration to be estimated at each time‐point. The HDO concentration in the body was also estimated from the ratio of the total imbibed D_2_O volume to an estimate of total body water.^32^

We used the image data to track the changes in ^2^H signal from different tissue compartments. The ^2^H images acquired at each time‐point were summed over the five echoes, and regions of interest (ROI) were then formed for a background region, general brain tissue, the lateral ventricles and for a region of high signal intensity thought to arise from blood vessels and CSF in the superior cistern. The SNR in images acquired before loading was too low to make good estimates of natural abundance signals, so values were normalized to the signal measured in the superior cistern ROI at the last time point of the initial loading period.

^2^H relaxation times for water in CSF, GM, and WM were calculated from data acquired during the six‐week loading period using the dual‐tuned ^2^H/^1^H coil. In each session, we acquired ^2^H 3D sagittal MEGE images (voxels = 6 × 6 × 10 mm^3^, FOV = 288 × 288 × 240 mm^3^, slices = 24) at a range of TR values, along with ^1^H 3D MEGE images (voxels = 3 ×3 × 5 mm^3^, FOV = 288 × 288 × 240 mm^3^, slices = 48, 15 echo times with TE_1_ = 2.5 ms, ΔTE = 2.34 ms, and TR = 41 ms). ^2^H MEGE data from Participants A and B were acquired with five echoes (TE_1_ = 4.3 ms, ΔTE = 8.4 ms), α = 60°, and TR = 68, 136, 272, 544 ms, with 8, 4, 2, and 1 averages, so that T_scan_ = 487 s per image. ^2^H MEGE data from participants C and D were acquired with six echoes (TE_1_ = 4.3 ms, ΔTE = 8.4 ms) and one additional TR‐value (TR = 816 ms, one average, T_scan_ = 730 s). The number of TE and TR values were increased to improve fitting quality. The ^2^H scanning sessions were performed twice on Participants C and D.

We also acquired ^1^H MPRAGE images (0.7 mm resolution) and ^1^H 3D MEGE images (3 × 3 × 5 mm^3^ voxels, 15 echo times, TE_1_ = 2.5 ms, ΔTE = 2.57 ms, and TR = 41 ms) from each participant in a separate scanning session using the Nova coil. These images were used for image segmentation and estimation of the ^1^H T_2_^*^ values.

For calculation of maps of ^2^H relaxation rate constants $R_{1}$ and R_2_^*^, we first estimated the variation of flip angle (α) over the image volume by summing the images across TEs, at each TR, and fitting the data voxel‐wise to a saturation recovery curve (i.e., fitting signal variation with TR for α, R_1_, and signal amplitude). The resulting flip‐angle maps were smoothed by averaging over 5 × 5 × 5 voxel neighborhood and the α‐values then used as fixed parameters in dual‐fitting the variation in signal intensity $S_{i,j}$ across ${\mathrm{TR}}_{i}$ and ${\mathrm{TE}}_{j}$ values for $R_{1}$, R_2_^*^ and signal amplitude, A. This involved minimisation of

(1) $$\sum_{i=1}^{n_{\mathrm{TR}}}\sum_{j=1}^{n_{\mathrm{TE}}}{\left\|\frac{A\sin\alpha \left(1-\exp\left(-R_{1}{\mathrm{TR}}_{i}\right)\right)}{1-\cos\;\alpha \;\exp\left(-R_{1}{\mathrm{TR}}_{i}\right)}*\exp\left(-R_{2}^{*}{\mathrm{TE}}_{j}\right)-S_{i,j}\right\|}^{2}$$

using the Matlab fmincon command. ^1^H R_2_^*^ maps were obtained by similar fitting to the exponential signal decay with TE in the ^1^H MEGE data acquired using the Nova coil.

To evaluate the relaxation times in different compartments, we segmented the ^1^H MPRAGE data (FSL FAST^33^) and transformed the resulting GM, WM, and CSF masks to the space of the ^2^H relaxation time maps. Following brain extraction (FSL BET^34^) and bias field correction, an affine matrix was obtained from image co‐registration (FSL FLIRT^35^, ^36^) that transformed the ^1^H MEGE data acquired using the Rapid Biomedical coil to the space of the ^1^H MEGE Nova Medical coil data, along with an affine matrix for the ^1^H MEGE to MPRAGE transformation. The MEGE data were summed across echoes and repetition times before co‐registration.

The brain‐extracted MPRAGE image was segmented to create binary masks for GM, WM, and CSF using FSL FAST.^33^ These masks were transformed to the ^2^H space using the previously obtained affine matrices and the outer regions of the CSF mask were manually removed so that the majority of the mask comes from the lateral ventricles. The new masks were applied to the relaxation maps for calculation of mean relaxation times for CSF, GM, and WM.

## 3 RESULTS

**FIGURE 1:**
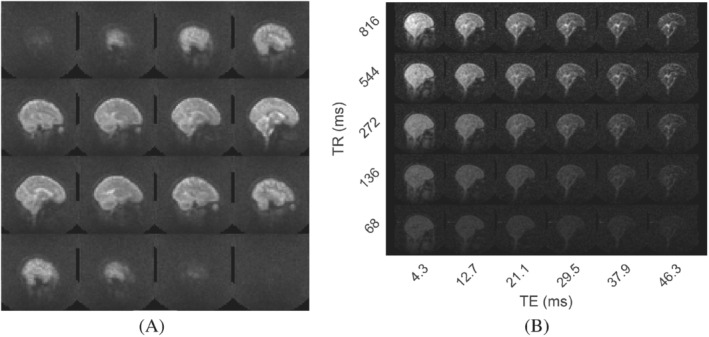
(A) 3D MEGE ^2^H image data from Participant C. Images produced by summing over six TE values and five TR values. (B) 3D MEGE ^2^H image from one slice from Participant D. Images are displayed with TE value varying horizontally and TR‐value varying vertically. Voxel size = 6 × 6 × 10 mm^3^, FOV = 288  × 288 mm^2^ in data used for both sub‐figures.

Figure 1 shows example ^2^H image data obtained during the steady‐state loading period. Figure 1A shows 3D sagittal image data produced by summing the MEGE data over TE and TR values. The resulting images clearly depict the brain anatomy and have a similar appearance to T_2_^*^‐weighted, ^1^H‐images. The CSF in the ventricles and at the cortical surface appears hyperintense, while regions where there is little partial‐voluming with CSF, such as the white matter in the corpus callosum, appear hypointense. Figure 1B shows the variation of image intensity with TE and TR in a central sagittal slice. The slower T_2_^*^ decay of the CSF signal compared with that of the GM and WM signals is evident, along with the signal saturation at reduced TR, and the reduction of contrast at low TE and TR values.

**FIGURE 2:**
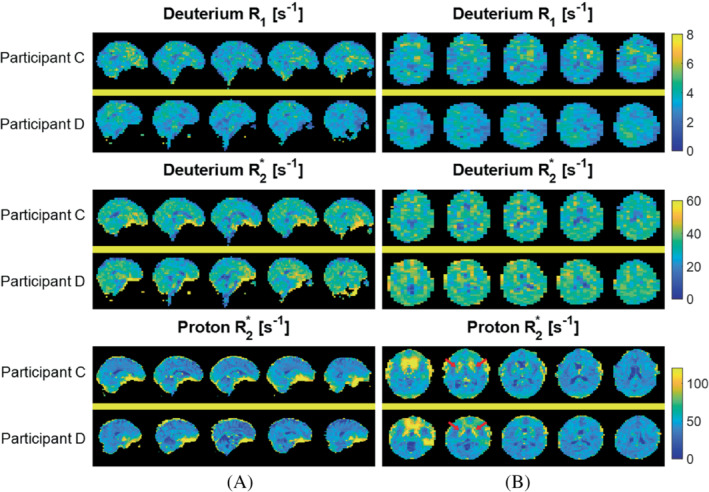
^2^H R_2_^*^ and R_1_ maps are shown along with ^1^H R_2_^*^ maps in sagittal (A) and axial (B) format. Maps show five central slices from Participants C and D. Relaxation maps were calculated from MEGE data equivalent to that displayed in Figure 1. The elevated R_2_^*^ in iron‐rich deep GM structures is evident in the lower slices of the ^1^H maps (red arrows), but is not seen in the ^2^H maps. The images shown have a reduced FOV of 204 × 204 mm^2^.

Figure 2 shows maps of the relaxation rate constants from two participants, with the dominant feature in the ^2^H maps being the reduced R_2_^*^ and R_1_ values in the ventricles. Table 1 reports the average and SDs of the ^2^H T_1_ and T_2_^*^ values, along with ^1^H T_2_^*^ values measured in GM, WM, and CSF in the four participants.

**TABLE 1:** Average and SD of ^2^H (T_2_^*^ and T_1_) and ^1^H (T_2_^*^) relaxation times in CSF, GM, and WM for different participants and visits

		Deuterium relaxation times [ms]						Proton relaxation times [ms]		
		CSF		GM		WM		CSF	GM	WM
Subject	Visit	T_1_	T_2_^*^	T_1_	T_2_^*^	T_1_	T_2_^*^	T_2_^*^	T_2_^*^	T_2_^*^
**A**	**1**	450 ± 200	110 ± 90	280 ± 100	32 ± 8	260 ± 100	30 ± 10	106 ± 90	26 ± 20	27 ± 20
**B**	**1**	520 ± 200	83 ± 50	300 ± 100	33 ± 10	280 ± 100	32 ± 20	103 ± 90	25 ± 20	23 ± 10
**C**	**1**	460 ± 100	76 ± 40	301 ± 80	31 ± 7	290 ± 100	30 ± 10	93 ± 100	22 ± 10	21 ± 6
**C**	**2**	390 ± 100	82 ± 60	295 ± 90	32 ± 8	267 ± 90	32 ± 10			
**D**	**1**	720 ± 200	84 ± 50	420 ± 100	31 ± 6	350 ± 100	28 ± 6	87 ± 90	23 ± 10	22 ± 8
**D**	**2**	510 ± 100	110 ± 40	320 ± 80	31 ± 6	277 ± 80	28 ± 6			
**Mean**		510	90	320	32	290	30	97	24	23
**SD**		100	10	50	1	30	1	5	2	2

*Note*: These values were produced by averaging over segmented relaxation time maps, similar to those shown in Figure 2. Average values and SDs across participants are also shown.

**FIGURE 3:**
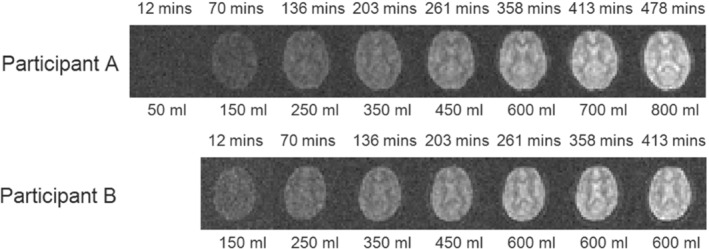
^2^H images acquired from two participants at different times during the initial, 8‐h heavy water loading period. The time since the first dose is indicated above each image and the cumulative dose of heavy water is indicated below. A single axial slice spanning the lateral ventricles is shown. The images shown are formed from the average over five echoes (TE_1_ = 8.9 ms, ΔTE = 8.4 ms). The images shown have a reduced FOV of 204  × 204 mm^2^.

**FIGURE 4:**
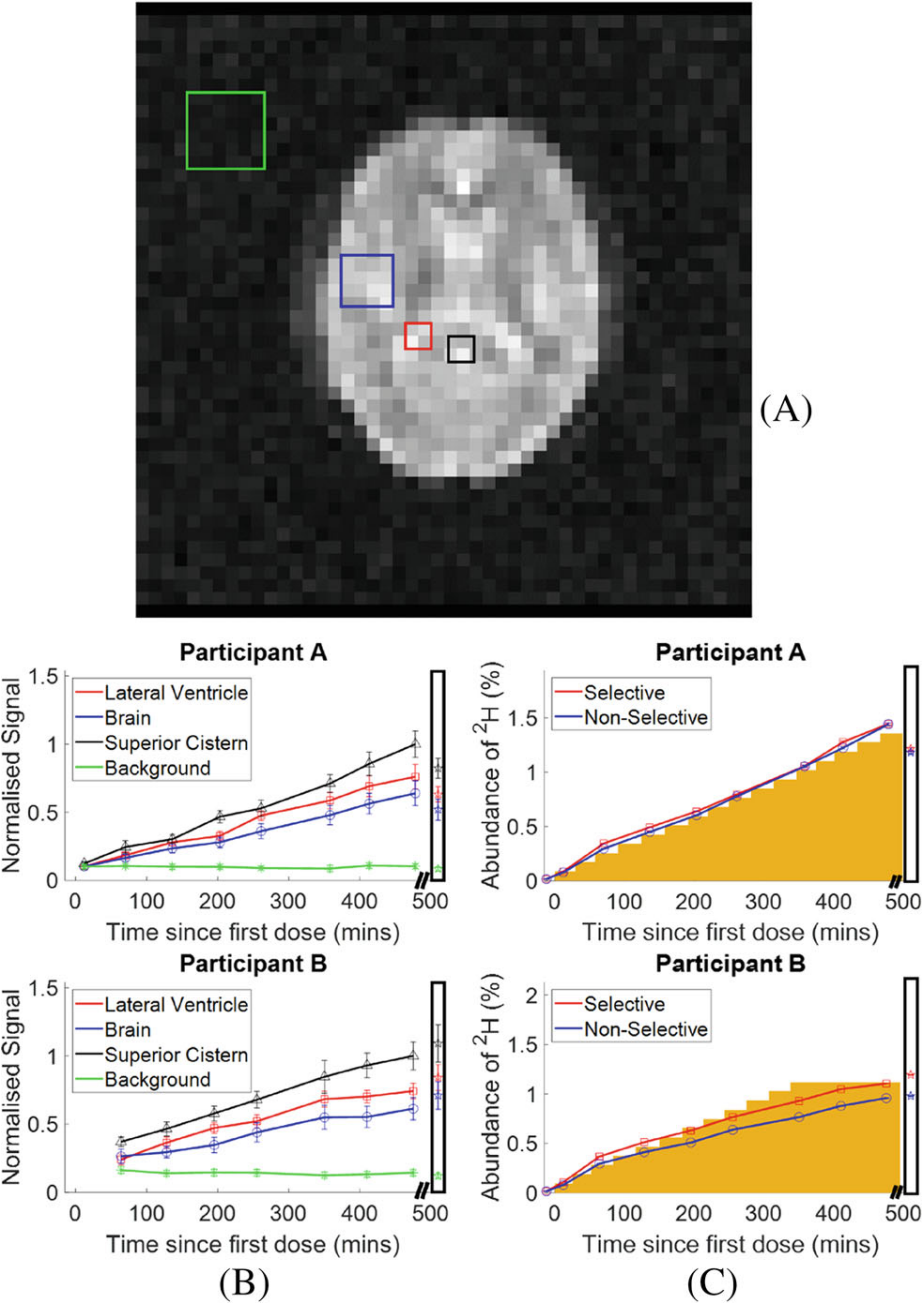
(A) Regions of interest used for following the time‐course of signal change during D_2_O loading. Black = superior cistern; Red = lateral ventricle; Blue = brain (GM, WM, and CSF); Green = background noise. (B) Time course of average signal change in image ROIs (red = lateral ventricle; blue = brain tissue; black = superior cistern; green = background noise) in two participants. Signals from all compartments are scaled by the superior cistern signal at the final measurement time‐point. Scaled signals measured at steady state (after 17‐days loading) are shown in the box at the far right (C) Time course of the concentration of deuterium in the brain estimated from the ^2^H spectroscopy measurements (red = from 2 cm slice at level of lateral ventricles; blue = whole head). Percentage estimated by scaling by the signal measured at natural abundance (assumed to be 0.015%). The orange blocks indicate the concentration estimated from the cumulative D_2_O dose and body weight. The measurements made at steady state (after 17 days of loading) are shown in the box at the far right.

Figure 3 reports example ^2^H images acquired from Participants A and B during the loading process. The different regions of interest in which the signal changes were tracked are indicated on Figure 4A (for Participant A) and Figure 4B shows the time courses from the different ROIs, along with the steady‐state values measured after 17 days of loading. Figure 4C plots the temporal variation of the absolute ^2^H concentration estimated from the spectroscopy measurements. The concentration calculated from the cumulative D_2_O dose and estimated total body water volume is shown for comparison.

## 4 DISCUSSION

The results shown in Figures 1 and 3 indicate that ^2^H images of 6  × 6  × 10 mm^3^ voxel size with a useful SNR can be acquired in 7.5 minutes at 7T with a head‐sized bird‐cage coil, when participants have been deuterium‐enriched to ˜1.5% concentration (˜100 times natural abundance). After summing over echo times these images (Figure 3) showed SNR ˜16 in brain tissue in the steady‐state condition (after 17 days of loading).

The measured relaxation times were reasonably consistent across the six measurements (Table 1), with CSF having significantly higher T_1_ and T_2_^*^ values than GM or WM (*p* < 0.007 for two‐sample t‐test). The measured T_1_ and T_2_^*^ values were consistently higher in GM than in WM, but the differences did not reach statistical significance (T_1_: *p* = 0.21; T_2_^*^: *p* = 0.08). The relatively coarse resolution of the ^2^H images made it difficult to avoid the effects of partial voluming, particularly of CSF and GM, and the limited range of TE (4.3–46.3 ms) and TR (68–816 ms) values reduced the accuracy of measurement of the long T_1_ and T_2_^*^ values in CSF, as is evident from the larger SDs of these measurements (Table 1). Longer echo trains with a duration that exceed the expected T_2_^*^ value and measurements at longer TR values should be used in future experiments targeting a better characterization of ^2^H HDO relaxation times in CSF. For example, simulations show that inclusion of an additional measurement with a TR of 1500 ms would halve the SD of the estimated T_1_ relaxation time of CSF but would also require 20 minutes of additional scanning time: use of an inversion recovery sequence may therefore be a better option. The average values of the relaxation times are consistent with values reported from non‐localized measurements of HDO signals in human,^2^, ^3^, ^11^ cat^27^ and rat^1^, ^2^ brain.

Focusing on human brain measurements, De Feyter et al.^2^ reported HDO T_1_ of 346 ± 5 ms at 4T, while Ruhm et al.^11^ measured 362 ± 6 ms at 9.4T – values which lie between the values for CSF (510 ms) and GM/WM (320/290 ms) measured here at 7T. As expected, the measured ^2^H T_1_‐values are significantly shorter than the corresponding ^1^H values at 7T,^32^ due to the quadrupolar relaxation of ^2^H. The long T_1_ of HDO in CSF relative to GM/WM will lead to greater saturation in the CSF signal in short CSI measurements used for DMI (for example Ruhm et al. used TR = 155 ms^11^) which needs to be considered when quantifying signals from other ^2^H‐labeled metabolites using natural abundance HDO signals. Bi‐exponential T_2_ decay was previously identified at 4T^2^ and 7T^37^ using non‐localized spin echo measurements: at 7T large (small) pools were found to have relaxation times of 29 ± 1 (412 ± 40) ms, respectively,^37^ consistent with our identification of short and long T_2_^*^ values in GM/WM (32/30 ms) and CSF (90 ms).

The TE‐summed MEGE images in Figures 1A and 3 show contrast that is dominated by T_2_^*^‐weighting, with the CSF appearing hyperintense relative to gray and white matter, as is the case in T_2_^*^‐weighted ^1^H images. A notable difference between the ^1^H and ^2^H R_2_^*^ maps (Figure 2) is that deep GM structures which appear with elevated R_2_^*^ in ^1^H maps due to their high iron content^38^ do not appear hyperintense in the ^2^H maps. This is a consequence of the dominance of quadrupolar, rather than dipolar, relaxation in the case of ^2^H and the relatively short T_2_ relaxation times that the quadrupolar interactions produce in tissue, along with the lower ^2^H gyromagnetic ratio. Together these mean that the large, microscopic and macroscopic field inhomogeneities generated by the iron‐rich inclusions in deep GM structures regions, which increase the ^1^H R_2_^*^ relaxation rate constants, do not have a significant effect on the measured ^2^H R_2_^*^‐values. The ^1^H R_2_^*^ maps also show larger regions of hyperintensity near the frontal sinuses due to the greater field‐inhomogeneity‐related intra‐voxel dephasing resulting from the higher γ of ^1^H. Signals from structures outside the brain (apart from the eyeball) are only evident in the ^2^H images acquired with the shortest TE value (Figure 1B) most likely because of the very short T_2_^*^ of HDO in muscle.^39^, ^40^

Figure 4 shows that the changes in HDO concentration during the initial heavy water loading could be readily tracked with imaging and spectroscopy. The concentrations estimated from the ^2^H spectra are in reasonably good agreement with the values calculated from the cumulative D_2_O dose and body mass (Figure 4C.) The signal amplitudes measured from ROIs in the brain images all have similar time‐courses and maintain relatively constant ratios, with values that are most likely dictated by differences in T_2_^*^‐weighting and water fraction in the different brain regions. This implies that the dispersal kinetics following oral ingestion of D_2_O are rapid throughout the body on the timescale of the measurements. This is consistent with previous measurements based on blood sampling which indicate that the half‐life of absorption into blood is ˜12 minutes, with similar time constants for dispersal into other body water compartments.^41^, ^42^ In our experiments the subject came out of the magnet bore between measurements, leading to the potential for changes in signal intensity due to variation of the slice position. Nevertheless the ^2^H signals tracked the monotonically increasing dose and the values measured at maximum dose were similar to those measured 17 days later during the steady state loading period. Although both participants had approximately the same weight and target D_2_O dose, Participant B was only able to ingest 600 ml during the initial loading. The deuterium concentration measured from Participant A was consequently higher at the end of the loading period. The rest of participant B's loading was completed over the following 4 days, along with the daily 50 ml top‐up and similar concentrations were measured from the two participants in the steady state (Figure 4C). The GCMS measurements of deuterium concentrations in the saliva samples from Participant A and B were 1.51% ± 0.09%, and 1.53% ± 0.17%, respectively.

Rapid increases in body water enrichment can lead to feelings of dizziness and nausea. These symptoms can occur at relatively low enrichments while equilibrium has not yet been achieved and are thought to result from temporary effects on the vestibular system due to density changes in the semi‐circular canals of the inner ear.^43^ Some participants experienced these effects and so the rate of D_2_O loading was slowed. The rapid loading was required for the parallel study, but a more gradual increase in heavy water uptake could be used for future MR‐loading experiments to minimize these effects.

## 5 CONCLUSIONS

Deuterium MR measurements at 7T have been successfully used to track the increase in concentration of ^2^H in brain during heavy water loading to 100 times natural abundance, in four human participants. Gradient echo images with an SNR of 16 and a voxel volume of 0.36 ml could be acquired in 7.5 minutes. ^2^H T_1_ and T_2_^*^ relaxation times from water in GM, WM, and CSF have also been measured at 7T. These relaxation times can be applied in research protocols using the natural abundance ^2^H signal from water for calibration. In future work we aim to track uptake from a single D_2_O dose on a shorter time scale, using faster, interleaved acquisition of ^2^H images and spectra.
